# Data-driven insights into predictors of stress and sleep health among Pakistani healthcare workers under rotational shifts

**DOI:** 10.1038/s41598-025-28872-z

**Published:** 2025-11-22

**Authors:** Sajeela Shahzad, Muhammad Awais Sattar, Farah Naeem Malik, Muhammad Aqib, Arooj Sattar

**Affiliations:** 1 Vital Signals Health, Inc., California, USA; 2 Automatic Control, Department of Computer Science, Electrical and Space Engineering, Luleå University of Technology, Luleå, Sweden; 3 District Headquarters Hospital, Attock, Pakistan; 4 Department of Gastroenterology and Dietics, I.M. Schenov First Moscow State Medical University, Moscow, Russia; 5 Department of Microbiology, Central Park Medical College, Lahore, Pakistan

**Keywords:** Perceived stress, Shift work disorder, Insomnia, Healthcare professionals, Multivariate analysis, Health care, Health occupations, Psychology, Psychology, Risk factors

## Abstract

Healthcare professionals in low- and middle-income countries often deal with persistent stress at work, driven by heavy workloads, irregular shift schedules, and limited institutional support. This cross-sectional study explored levels of stress, emotional well-being, sleep issues, and shift work-related challenges among healthcare workers in Pakistan. Overall, participants reported moderate levels of stress and sleep disturbances. Interestingly, positive emotions outweighed negative ones. Age stood out as the strongest predictor of stress; those in their mid to late careers experienced significantly higher stress levels. While female participants tended to report more stress than males, the difference wasn’t statistically significant. Marital status and exposure to secondhand smoke were linked to higher stress in univariate analyses, and higher body weight showed a slight association in adjusted models. However, factors like shift type and the number of weekly working hours didn’t significantly predict stress. Notably, perceived stress was a strong independent predictor of negative emotional states, even after accounting for past mental health issues and work-related injuries. Age emerged as the strongest predictor of stress, with mid- and late-career professionals reporting significantly higher levels than younger colleagues. These findings emphasize the need for age-sensitive mental health interventions and stress management strategies in healthcare settings.

## Introduction

Healthcare workers (HCWs) routinely work in high-pressure environments, facing long hours, emotionally intense patient interactions, and urgent, often critical decision-making. These ongoing demands can lead to mental and physical exhaustion, increasing the risk of psychological distress and burnout, which is typically marked by emotional fatigue, detachment from patients, and a reduced sense of accomplishment^1,2^. In countries like Pakistan, where healthcare systems face significant resource limitations, these pressures are intensified by staff shortages, limited infrastructure, and a lack of accessible mental health services^3,4^. These combined pressures place Pakistan’s healthcare workforce under sustained psychological strain. Furthermore, the country’s ethnic and cultural diversity brings additional complexity, as coping styles and stress responses may vary across different groups^5^. These cultural and interpersonal variations highlight the need to interpret occupational stress within the broader social context of Pakistani healthcare. Although collectivism was not directly measured, the interpretation of results considers Pakistan’s collectivist social context, where interdependence and family support often moderate stress experiences. These complex realities necessitate a multidimensional lens to understand healthcare workers’ well-being. In low- and middle-income countries (LMICs) such as Pakistan, stress among healthcare workers is shaped not only by heavy workloads but also by chronic shortages of staff and resources. This perspective connects institutional pressures with the lived psychological experiences of HCWs, underscoring the need for targeted, context-specific research. The biopsychosocial model emphasizes the interplay of biological, psychological, and social factors in health and disease^6^. This model is particularly relevant for understanding burnout among healthcare workers (HCWs), a significant issue globally.While widely applied in international studies, its specific relevance to Pakistani healthcare workers has been understudied. Understanding this link within Pakistan’s sociocultural and institutional context addresses a major gap in existing literature. This theoretical perspective provides a foundation for examining how biopsychosocial interactions manifest uniquely within Pakistan’s healthcare infrastructure. Despite numerous global studies on occupational stress and sleep health, few have examined how these dynamics operate within Pakistan’s healthcare system, which faces distinct structural and cultural challenges. The scarcity of empirical data integrating psychosocial models with shift-related stress metrics among Pakistani healthcare professionals underscores the novelty of this investigation. Burnout in healthcare is a syndrome characterized by emotional exhaustion, depersonalization, and reduced personal accomplishment, stemming from chronic workplace stress. A meta-analysis found a high prevalence of burnout among primary HCWs in low- and middle-income countries^7^. Factors contributing to burnout include work demands, organizational issues, and psychological stress^8,9^. The COVID-19 pandemic exacerbated burnout rates, with prevalence ranging from 4.3% to 90.4% among HCWs^10^. Burnout is also a concern in South Asian settings, including Pakistan^7^. Coping strategies and social support play crucial roles in managing stress and trauma^11^. Addressing burnout requires both individual and system-level interventions to promote resilience and improve working conditions^12^. Collectively, these findings indicate that both organizational and individual factors contribute to occupational stress, highlighting the need for integrated intervention strategies. Building on this evidence, it is important to explore how these mechanisms interact to shape the persistence of stress among HCWs. Stress in healthcare is rarely caused by a single factor. It often results from a combination of urgent clinical situations, heavy administrative loads, emotional demands, and unpredictable shift schedules^6^. Among these overlapping pressures, shift work has emerged as one of the most influential determinants of stress and sleep disturbance. Lazarus’s transactional model suggests that how a person evaluates and responds to stressors plays a central role in how they experience stress^13^. In clinical practice, HCWs frequently encounter high patient volumes, emotionally challenging scenarios, and erratic schedules^14–16^. In Pakistan, nearly 68% of HCWs report moderate to severe stress, but fewer than one in five seek formal help largely due to stigma, poor access, and concerns about professional consequences^17^. Cultural values that emphasize endurance and silence about distress often discourage help-seeking^18^. Gender also plays a role: women often balance demanding shifts with family responsibilities, while men may face stress related to workplace expectations and public scrutiny^19^. Beyond these psychosocial stressors, the structure and timing of work schedules themselves pose unique physiological and emotional challenges. Among the many contributors to stress, shift work stands out. Night shifts in particular are linked to disrupted circadian rhythms, sleep loss, cognitive decline, and a higher risk of chronic illnesses^20^. The impact of shift work extends beyond fatigue. It has been associated with insomnia, mood disorders, substance use, and even suicidal ideation in extreme cases^21^. Shift Work Sleep Disorder (SWSD), defined by excessive sleepiness and difficulty sleeping during rest hours, is a recognized condition caused by these circadian disruptions^22^. The interrelationship between stress and sleep is well-known: chronic stress enhances physiological hyperarousal, impairing sleep quality, while sleep deficiency amplifies stress responses^23^. Female HCWs working on night shifts may be especially vulnerable to depression due to both hormonal and social influences^24^. Healthcare workers suffering from poor sleep often report difficulty focusing, low energy, and trouble with memory or decision-making. These issues can directly impact patient care. Sleep-deprived HCWs are more likely to experience burnout, irritability, job dissatisfaction, and absenteeism^25^. In Pakistan’s already stretched healthcare system, the cognitive effects of sleep deprivation, including delayed reaction times, pose serious safety concerns^26^. These safety risks underscore how physiological fatigue interacts with psychological strain, further reinforcing the need for preventive measures within shift-based systems. Additionally, shift workers are more likely to adopt unhealthy coping habits such as smoking, sedentary behavior, and poor diets. These behaviors increase the risk of obesity, metabolic dysfunction, and endocrine imbalance. Alterations in cortisol levels are common in shift workers. Both night and rotating shift workers show signs of chronic stress through elevated cortisol levels and abnormal cortisol awakening responses (CAR), even during rest periods. This physiological dysregulation is thought to result from long-term circadian misalignment and poor sleep^27^. These patterns may help explain why shift work is linked to a broad range of health issues, including gastrointestinal disorders, breast cancer, cardiovascular disease, and sleep apnea^28^. Recent systematic reviews highlight the significant impact of shift work on sleep, mental health, and physical well-being. Night shifts and rotating schedules are associated with increased sleep disturbances, fatigue, and health risks^29,30^. Shift workers struggle to balance sleep needs with other responsibilities, often prioritizing family over rest^31^. Metabolic alterations, including impaired glycemic control and cholesterol imbalances, are observed in shift workers^32^. Various interventions, such as light therapy and cognitive behavioral approaches, show moderate effectiveness in managing sleep issues^33^. The prevalence of sleep disorders and poor sleep quality is high among specific occupations like healthcare, firefighters and aircrew^34,35^. Additionally, poor sleep quality in adolescents is associated with decreased school engagement and performance^36^, highlighting the broader societal impact of sleep health. Considering these interlinked biological, psychological, and occupational factors, a systematic evaluation of stress and sleep health among Pakistani HCWs is both timely and necessary. The present study therefore aims to quantify these relationships using validated psychometric tools and multivariate analysis. This study uses four validated tools to explore stress and sleep-related outcomes among Pakistani HCWs–the Perceived Stress Scale (PSS), Positive and Negative Affect Schedule (PANAS), Ford Insomnia Response to Stress Test (FIRST), and the Shift Work Disorder Index (SWDI)–providing a structured assessment of perceived stress, emotional states, vulnerability to stress-induced insomnia, and symptoms related to shift work. Taken together, these perspectives frame our two guiding research questions: (1) Do different shift schedules significantly affect perceived stress and sleep health? and (2) Are there demographic differences in vulnerability to stress among Pakistani HCWs?. Together, these instruments provide a structured assessment of perceived stress, emotional states, vulnerability to stress-induced insomnia, and symptoms related to shift work. The PSS measures how unpredictable and overwhelming individuals perceive their lives to be^37^. PANAS evaluates both positive and negative emotional states and is widely used in psychological and clinical studies^38,39^. The FIRST identifies individuals prone to insomnia when under stress^40,41^. The SWDI is designed to screen for symptoms such as poor sleep quality, anxiety, and depressive features specific to shift work^42^. While the broader conceptual model included cognitive and cultural dimensions, the present analysis concentrates on three core outcomes: perceived stress, insomnia vulnerability, and shift-work-related dysfunction. Cognitive performance will be examined in future studies using objective testing. Informed by existing literature and the unique working conditions in Pakistan, this study proposes the following hypotheses:

- Night shift workers will report higher levels of stress, insomnia, and symptoms of SWSD due to circadian disruption.
- Female HCWs will experience greater emotional exhaustion and higher stress levels compared to males, shaped by both biological and social roles.
- Professionals in the 26–45 age range will report the highest stress, likely reflecting increased job demands and family responsibilities.
- Ethnic identity will influence stress levels, with collectivist cultures potentially offering stronger social support and resilience.
- Extended exposure to shift work will impair cognitive performance and elevate the risk of workplace accidents.
- Individuals diagnosed with SWSD will show poorer mental health and higher stress levels than those without the disorder.

This study aims to fill critical gaps in the literature by investigating the interplay of occupational stress, sleep disorders, emotional regulation, and cultural context among HCWs in Pakistan. These hypotheses directly align with our study objectives: first, to identify demographic and occupational predictors of stress; and second, to evaluate how shift-related variables contribute to sleep-related stress dysfunction.

## Methodology

### Study design and participants

This prospective cross-sectional study was conducted from June to August 2024 at Asfandyar Bukhari District Hospital, Attock, Pakistan. Participants were recruited through a convenience sampling method, using both in-person distribution of paper-based surveys during routine clinical shifts and an online survey form. All participants received a detailed informed consent form at the beginning of the survey, whether in paper or digital format. The form outlined the study’s objectives, procedures, potential risks, benefits, and the voluntary nature of participation. Consent was considered obtained when participants, after reading the form, chose to proceed with completing the survey. No personally identifiable information was collected, and all responses were kept anonymous. Eligible participants included physicians, pharmacists, nurses, dentists, and allied health professionals. Informed consent was obtained electronically from all participants prior to data collection. Each participant was presented with a comprehensive consent form outlining the study objectives, procedures, and voluntary nature of participation, and consent was implied by their decision to proceed with the survey. The study protocol was approved by the hospital’s Departmental Ethical Committee and was conducted according to the principles outlined in the Declaration of Helsinki. The use of convenience sampling and self-reported questionnaires may introduce selection and response biases, which should be considered when interpreting the findings.

### Sample size estimation

The required sample size was calculated using the standard formula for estimating proportions, as shown in Eq. (1):

1 $$\begin{aligned} n = \frac{Z^2 \cdot p(1 - p)}{d^2} \end{aligned}$$

where:

- *n* = required sample size
- *Z* = Z-score corresponding to the desired confidence level (e.g., 1.96 for 95%)
- *p* = estimated proportion of the population (prevalence)
- *d* = margin of error (precision)

Based on this equation, a sample size of 200 was calculated and used.

### Instruments and measures

Participants completed four validated instruments to assess psychological and sleep-related variables:

- *Perceived stress scale (PSS):* Measures perceived unpredictability and lack of control in life.
- *Positive and negative affect schedule (PANAS):* Assesses emotional states using two subscales (positive and negative affect).
- *Ford insomnia response to stress test (FIRST):* Evaluates individual susceptibility to stress-related insomnia.
- *Shift work disorder index (SWDI):* Screens for symptoms related to Shift Work Sleep Disorder.

Each instrument used in this study has demonstrated strong reliability and validity across diverse populations. The Perceived Stress Scale, Positive and Negative Affect Schedule, Ford Insomnia Response to Stress Test, and Shift Work Disorder Index are all well-established tools for assessing psychological and sleep-related outcomes. In the current sample, all instruments exhibited satisfactory internal consistency, with Cronbach’s alpha values ranging from 0.747 to 0.815. Operational definitions were applied to ensure clarity and reproducibility. ”Extended exposure to shift work” refers to continuous employment in rotating or night shifts for six months or longer; ”poor mental health history” denotes any self-reported diagnosis or treatment for anxiety, depression, or stress-related disorders; and ”cultural support” represents perceived emotional or practical assistance derived from family or peer networks.

### Data collection procedures

Of the total responses, 28 were received electronically, while the remainder were completed on paper during working hours. Participants were instructed to complete the forms independently. The questionnaires were self-administered and collected under supervision to ensure completeness and accuracy. Anonymity and confidentiality were maintained throughout the process.

### Statistical analysis

Categorical variables were summarized as frequencies and percentages. Continuous variables were evaluated for normality using visual plots and the Shapiro–Wilk test. Descriptive statistics included means, standard deviations, medians, and interquartile ranges.Internal consistency for each scale was assessed using Cronbach’s alpha, with values above 0.70 considered acceptable. Pearson and Spearman correlation analyses were conducted to examine relationships among continuous variables. Between-group comparisons of stress scores were performed using independent t-tests or one-way ANOVA, followed by Tukey’s HSD for post hoc analysis when appropriate. Multivariate linear regression was applied to identify independent predictors of perceived stress. An additional model assessed the influence of stress on negative affect while controlling for mental health history and occupational injury. All analyses were conducted using Python 3.13, with statistical significance set at $\alpha = 0.05$. One-way ANOVA was used to examine mean stress differences across categorical variables such as age and gender, followed by Tukey’s HSD post hoc tests. Multiple linear regression identified independent predictors of perceived stress while controlling for confounders. The final sample size (n = 200) provided roughly 80% power to detect medium effect sizes.

### Control of confounders

Although no experimental control groups were used, potential confounding variables such as age, gender, job role, and shift type were accounted for using multivariate regression techniques.

## Results

### Descriptive statistics and internal consistency of psychometric scales

Descriptive statistics were calculated for the Perceived Stress Scale (PSS), Ford Insomnia Response to Stress Test (FIRST), Positive and Negative Affect Schedule (PANAS), and Shift Work Disorder Index (SWDI). Total scores were derived by summing Likert-scale responses according to each tool’s standard protocol. Table 1 summarizes the mean, standard deviation, and distribution percentiles for each psychometric instrument. The PSS had a mean score of 17.89 (SD = 6.78), indicating moderate perceived stress levels. FIRST showed a mean of 19.15 (SD = 5.81), reflecting moderate susceptibility to insomnia under stress. PANAS-PA yielded higher positive affect (M = 29.87, SD = 7.24) compared to PANAS-NA (M = 23.29, SD = 7.61), while SWDI showed a moderate presence of shift work-related symptoms (M = 7.01, SD = 4.70).

**Table 1:** Descriptive statistics for psychometric scale scores.

Scale	Mean	SD	Min	25%	Median	75%	Interpretation
PSS	17.89	6.78	0	15	19	22	Moderate stress, variable
FIRST	19.15	5.81	9	15	19	23	Moderate sleep disturbance
PANAS-PA	29.87	7.24	10	26	30	35	Emotional resilience retained
PANAS-NA	23.29	7.61	10	17.75	23	28	Varies across individuals
SWDI	7.01	4.70	0	4	7	11	Symptoms are present in a subset

### Internal consistency of psychometric instruments

Internal consistency of the psychometric tools was evaluated using Cronbach’s alpha ($\alpha$), with $\alpha \ge 0.70$ considered acceptable. All instruments demonstrated acceptable to strong reliability, as shown in Table 2. PANAS-NA had the highest reliability ($\alpha = 0.815$), followed by FIRST ($\alpha = 0.783$).

**Table 2:** Internal consistency (Cronbach’s Alpha) of psychometric scales used in the study.

Instrument	Cronbach’s Alpha
PSS (Perceived Stress)	0.758
FIRST (Insomnia Tendency)	0.783
PANAS-PA (Positive Affect)	0.758
PANAS-NA (Negative Affect)	0.815
SWDI (Shift Work Disorder)	0.747

### Intercorrelations among psychometric measures

Pearson correlation coefficients were calculated to examine relationships among PSS, FIRST, PANAS-PA, PANAS-NA, and SWDI scores (Table 3). Perceived stress (PSS) showed moderate positive correlations with insomnia vulnerability (FIRST; $r = 0.351$), negative affect (PANAS-NA; $r = 0.305$), and positive affect (PANAS-PA; $r = 0.314$), suggesting that higher stress levels are linked with emotional and sleep-related difficulties.Insomnia (FIRST) was strongly associated with negative affect ($r = 0.522$) and modestly with shift work symptoms (SWDI; $r = 0.289$). SWDI was weakly correlated with PSS ($r = 0.278$) and PANAS-NA ($r = 0.281$). PANAS-PA and PANAS-NA were only weakly related ($r = 0.211$), supporting their conceptual independence.

**Table 3:** Correlation matrix among stress, sleep, mood, and work disruption scales.

Scale	PSS	FIRST	PA	NA	SWDI
PSS	1.000	0.351	0.314	0.305	0.278
FIRST	0.351	1.000	0.064	0.522	0.289
PANAS-PA	0.314	0.064	1.000	0.211	0.149
PANAS-NA	0.305	0.522	0.211	1.000	0.281
SWDI	0.278	0.289	0.149	0.281	1.000

### Gender differences in perceived stress

**Fig. 1:**
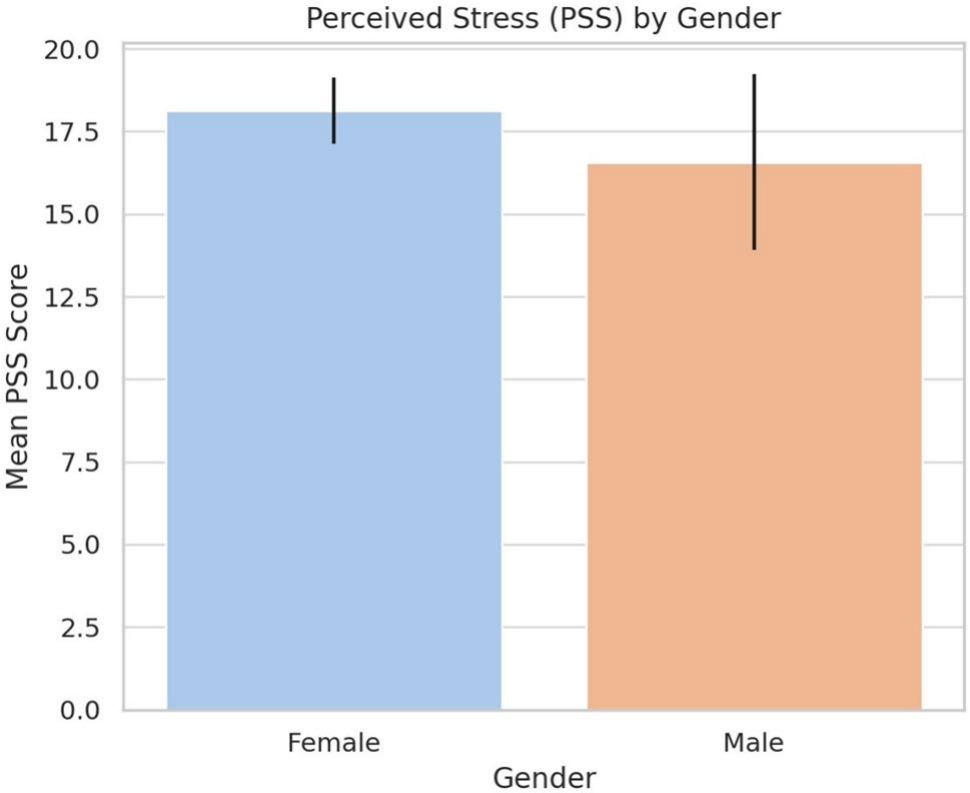
Perceived stress (PSS) by gender.

An independent samples *t*-test with Welch’s correction compared mean PSS scores by gender. As shown in Fig. 1, females reported slightly higher stress ($M = 18.12$) than males ($M = 16.57$), but the difference was not statistically significant ($t = -1.07$, $p = 0.292$). Overlapping confidence intervals suggest no meaningful gender-based difference in perceived stress.

### Age differences in perceived stress

**Fig. 2:**
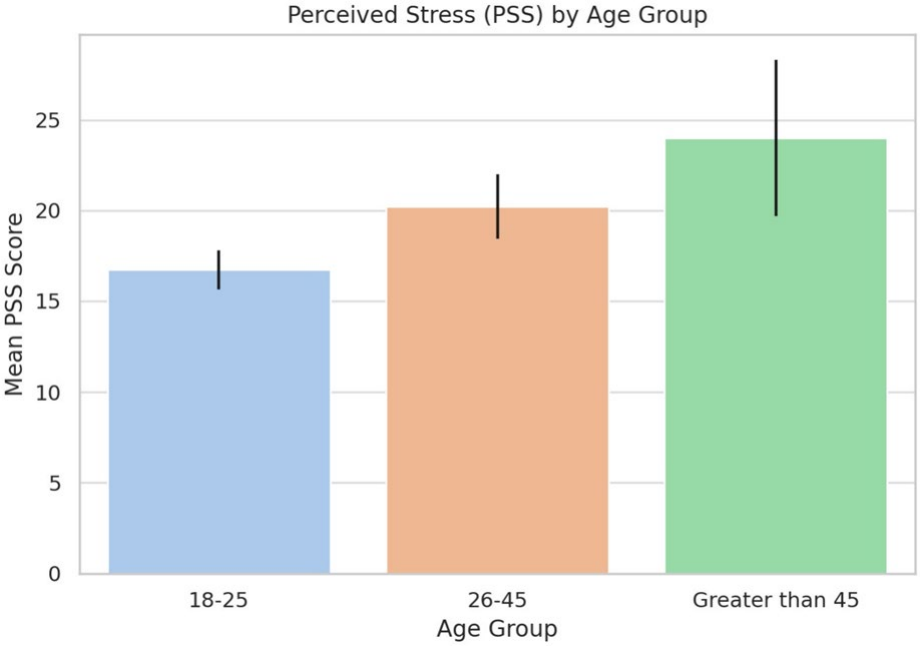
Perceived stress stress (PSS) by age group.

A one-way ANOVA compared PSS scores across age groups (18–25, 26–45, >45). As shown in Table 4 and Fig. 2, stress levels increased with age ($p < 0.001$), highest in the >45 group ($M = 24.00$), followed by 26–45 ($M = 20.22$), and 18–25 ($M = 16.72$). Due to the small size of the >45 group, results should be interpreted cautiously. Post hoc Tukey’s test (Table 5) showed significantly higher stress in the 26–45 group compared to 18–25 ($p = 0.002$); other comparisons were not significant.

**Table 4:** One-way ANOVA results: mean perceived stress scores by age group.

Age group	Mean PSS	95% CI
18–25 years	16.72	± 1.08
26–45 years	20.22	± 1.77
>45 years	24.00	± 4.31

**Table 5:** Post-hoc comparison of perceived stress across age groups (Tukey HSD).

Comparison	Mean Difference	p-adj	95% CI	Significant
18–25 vs. 26–45	3.50	0.002	[1.07, 5.93]	Yes
18–25 vs.>45	7.28	0.076	[–0.59, 15.14]	No
26–45 vs. >45	3.78	0.508	[–4.24, 11.79]	No

### Marital status and stress

**Fig. 3:**
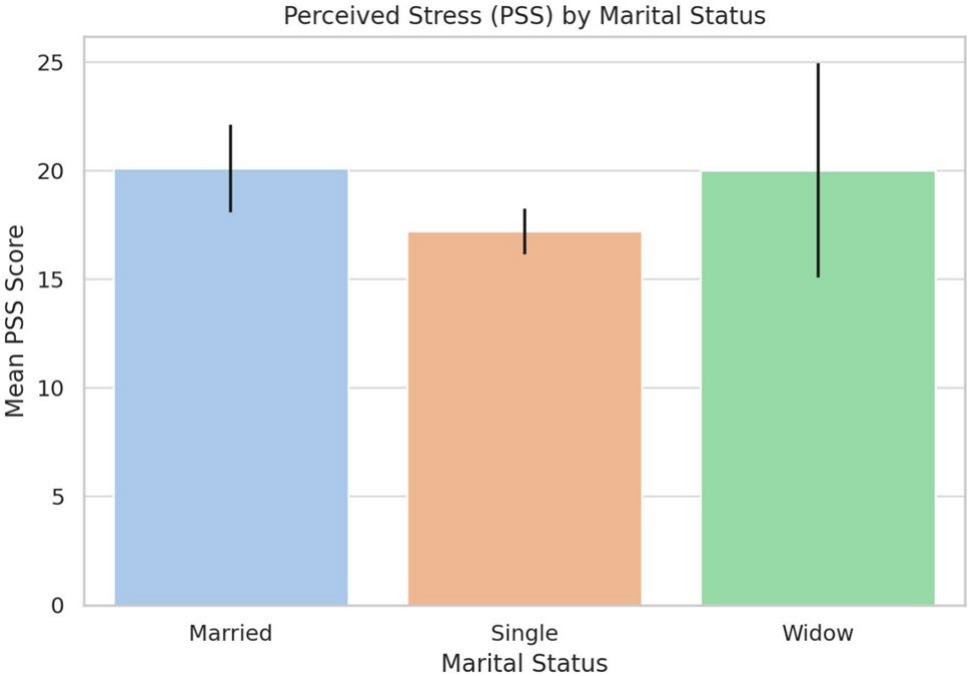
Perceived stress stress (PSS) by marital status.

A one-way ANOVA assessed PSS differences across marital status groups. As shown in Table 6 and illustrated in Fig. 3, stress levels varied significantly ($p = 0.035$). Married participants reported higher stress (mean = 20.09) than singles (mean = 17.19). Widowed participants had a similar mean (mean = 20.00), but a wide confidence interval limits interpretation due to the small sample size.

**Table 6:** Mean perceived stress scores by marital status.

Marital status	Mean PSS	95% CI
Single	17.19	± 1.06
Married	20.09	± 2.00
Widowed	20.00	± 4.93

### Smoking status and stress

**Fig. 4:**
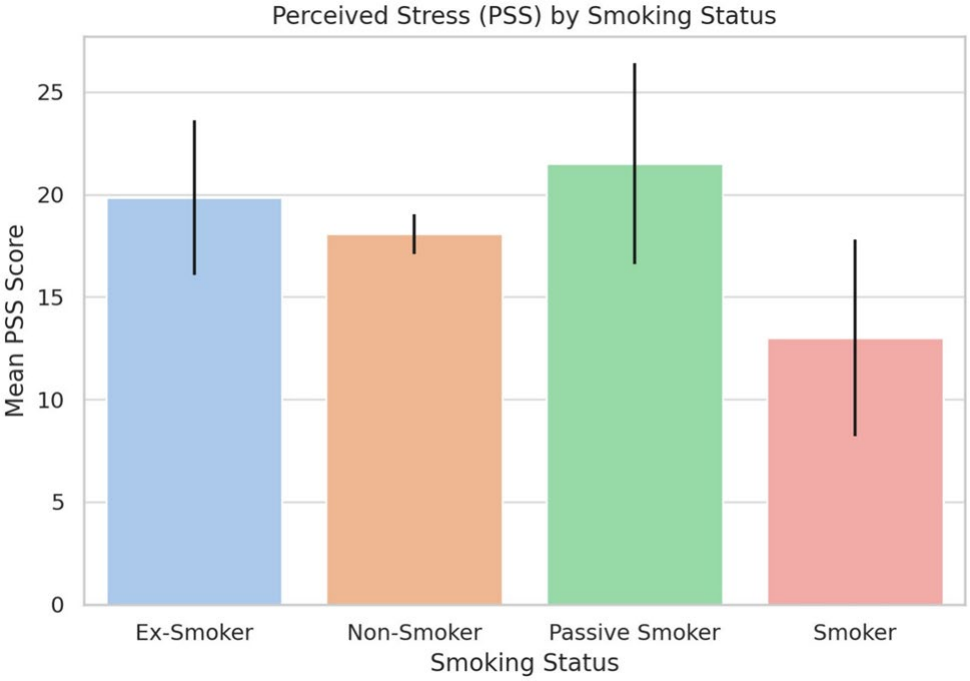
Perceived stress (PSS) by smoking status.

A one-way ANOVA compared PSS scores across smoking groups: current, ex-, passive, and non-smokers. As shown in Table 7, and illustrated in Fig. 4 stress differed significantly by smoking status ($p<$ 0.05). Passive smokers reported the highest stress (M = 21.50), followed by ex-smokers (M = 19.83), non-smokers (M = 18.07), and current smokers (M = 13.00). Wide confidence intervals in some groups reflect small sample sizes.

**Table 7:** Mean perceived stress scores by smoking status.

Smoking status	Mean PSS	95% CI
Smoker	13.00	± 4.81
Ex-Smoker	19.83	± 3.77
Passive Smoker	21.50	± 4.90
Non-Smoker	18.07	± 0.98

### Substance use and perceived stress

**Fig. 5:**
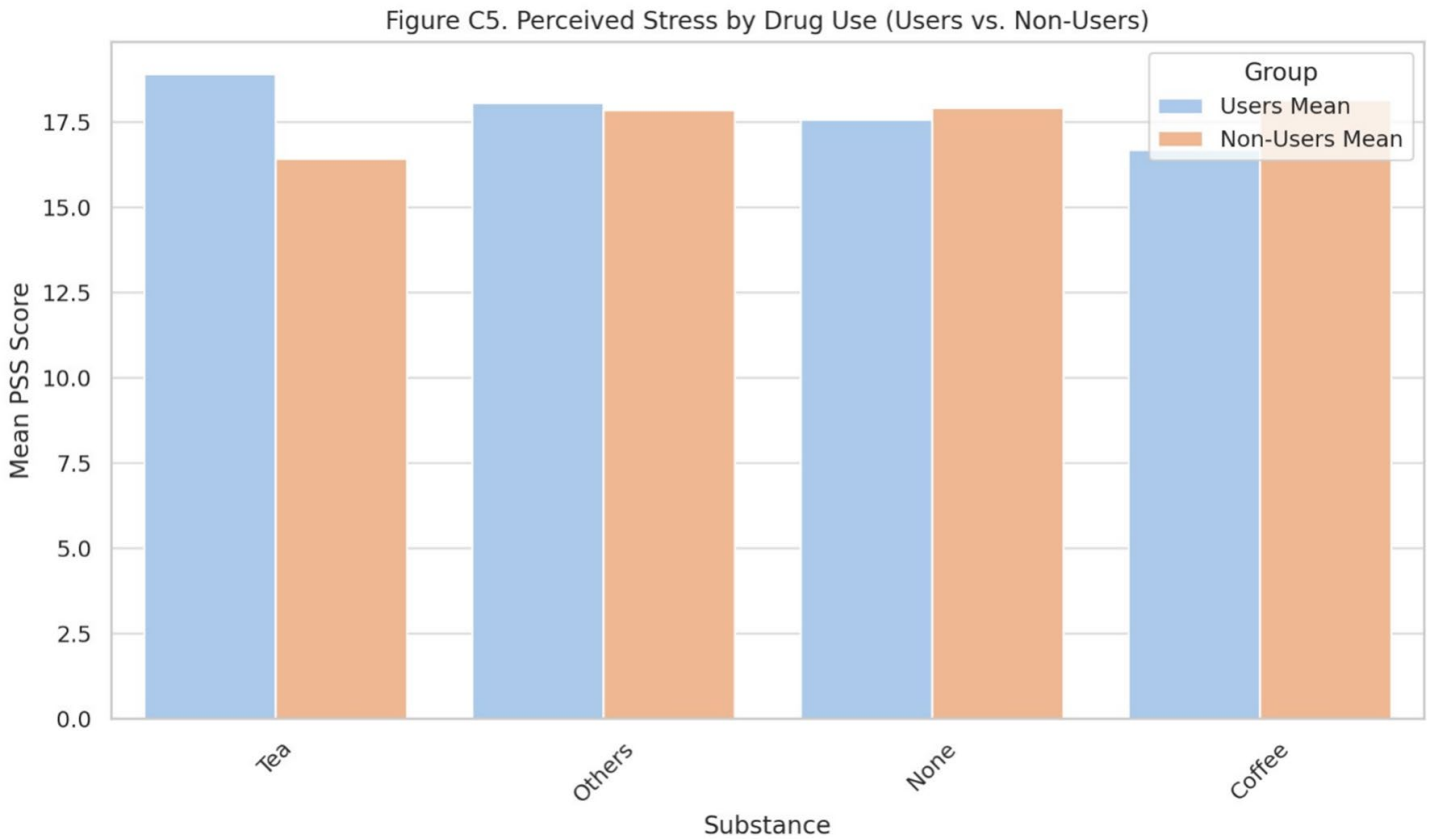
Perceived stress by drug use.

Exploratory comparisons of PSS scores by substance use (tea, coffee, tobacco, marijuana, cigarettes, stimulants) are shown in Table 8, and illustrated in Fig. 5. Tea users (n = 117) reported slightly higher stress (M = 18.91) than non-users (M = 16.43). Tobacco and cigarette users showed lower stress, but subgroup sizes were very small. Marijuana and stimulant use were reported by only one participant each. Findings are preliminary due to limited sample sizes.

**Table 8:** Mean perceived stress scores among substance users versus non-users.

Substance	Users’ mean PSS	Non-users’ mean PSS	Users (n)
Marijuana	20.00	17.87	1
Prescription CNS stimulants	20.00	17.87	1
Tea	18.91	16.43	117
Tobacco	14.75	17.95	4
Cigarette	11.83	18.07	6

### Body weight and stress

**Fig. 6:**
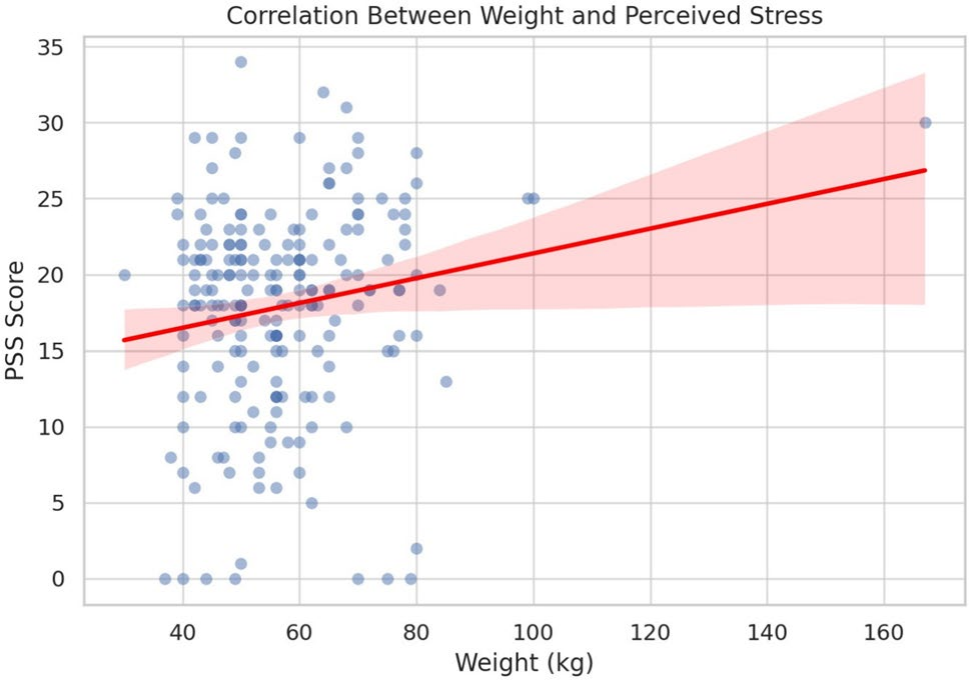
Correlation between body weight and perceived stress.

Correlations between body weight and PSS are shown in Table 9 and Fig. 6. Pearson’s *r* indicated a weak but significant positive association ($r = 0.174$, $p = 0.014$), while Spearman’s $\rho$ showed a similar, non-significant trend ($\rho = 0.133$, $p = 0.061$). These results suggest a modest link between higher weight and stress.

**Table 9:** Correlation between body weight and perceived stress.

Statistic	Value	p-value
Pearson *r*	0.174	0.014
Spearman $\rho$	0.133	0.061

### Shift type and stress

**Fig. 7:**
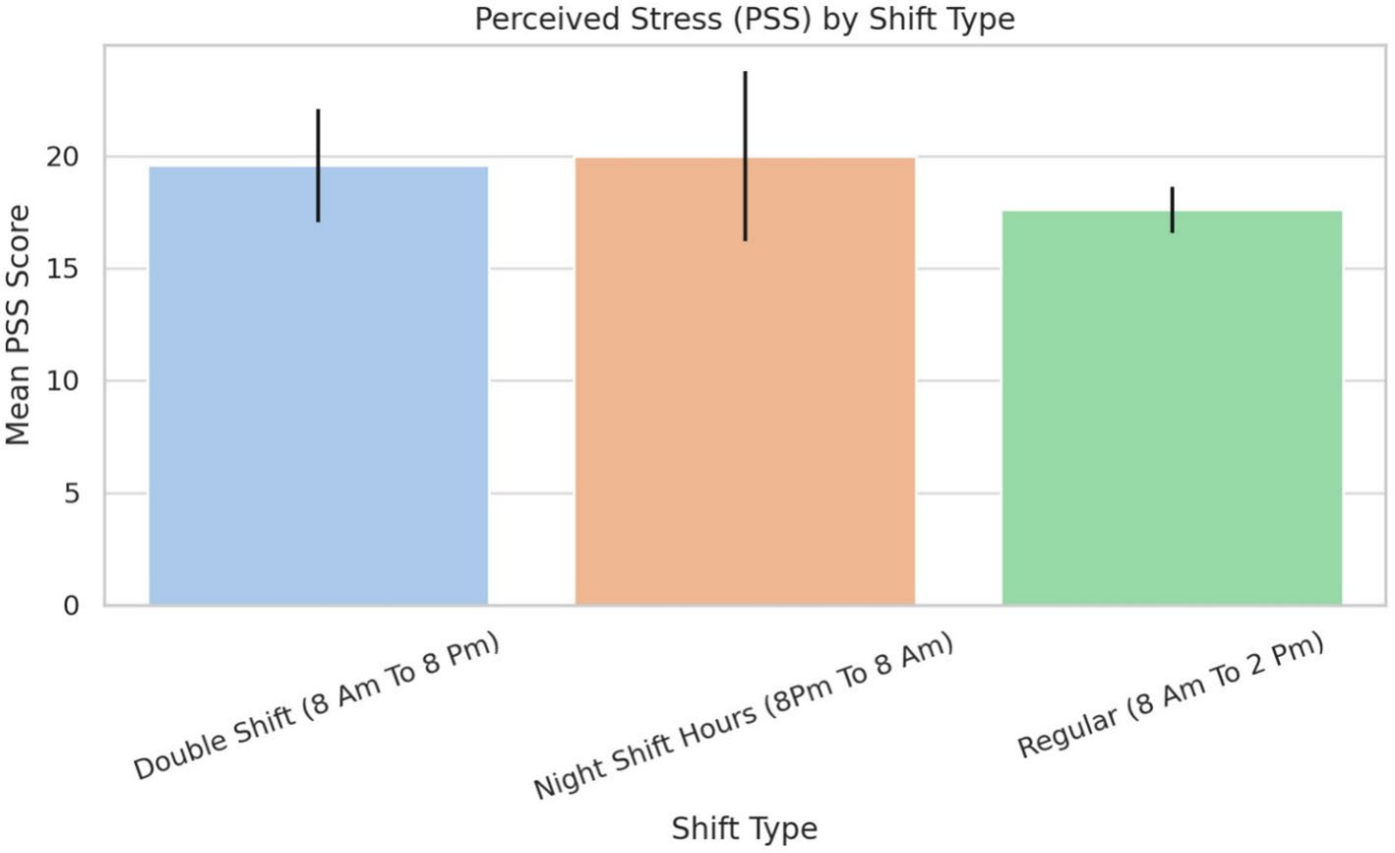
Perceived stress (PSS) by shift type.

A one-way ANOVA assessed PSS differences across shift types: regular, double, and night. As shown in Table 10 and Fig. 7, night shift workers had the highest stress (M = 20.00), followed by double (M = 19.59) and regular shifts (M = 17.61). However, the differences were not statistically significant ($p = 0.33$), with wide confidence intervals indicating variability.

**Table 10:** Mean perceived stress scores by shift type.

Shift type	Mean PSS	95% CI
Regular (8 AM–2 PM)	17.61	± 1.03
Double Shift (8 AM–8 PM)	19.59	± 2.52
Night Shift (8 PM–8 AM)	20.00	± 3.78

### Shift duration and perceived stress

**Fig. 8:**
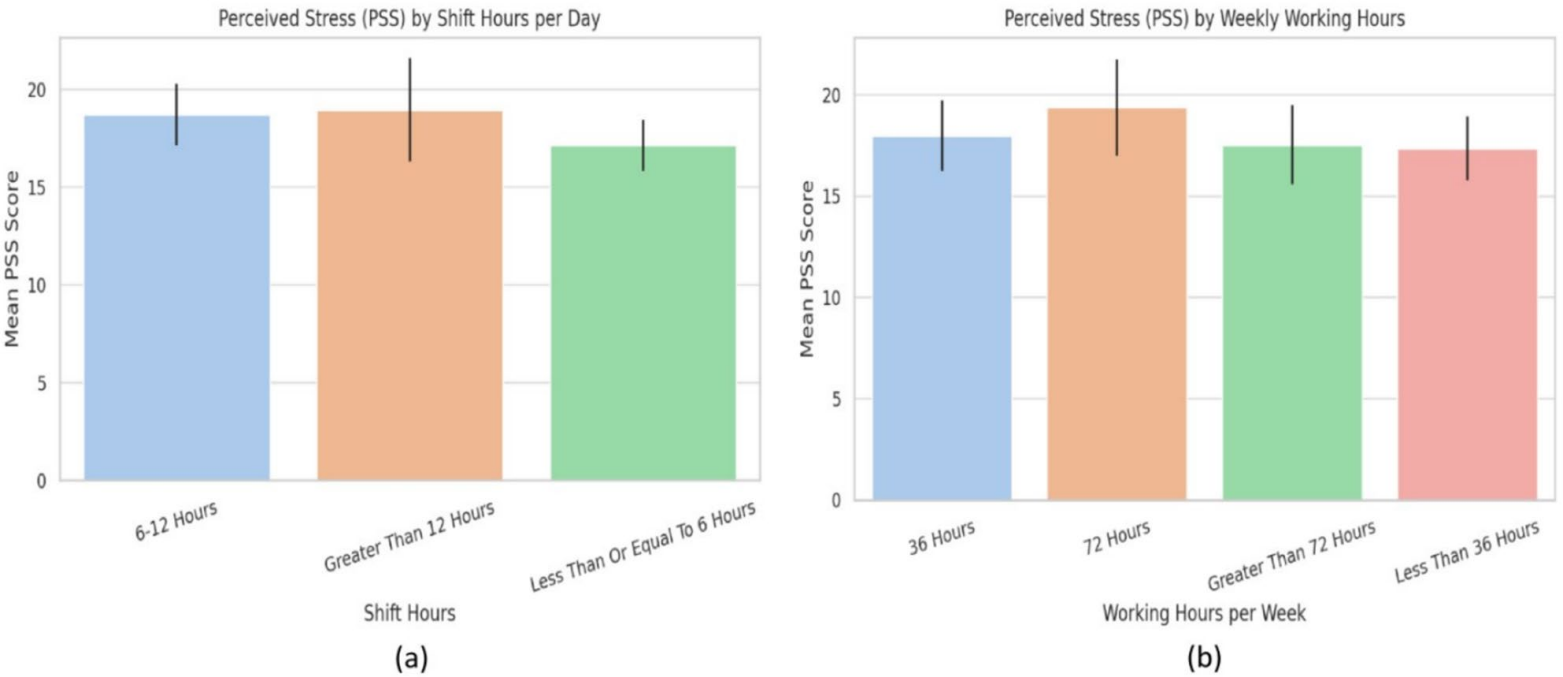
**a** Perceived stress (PSS) by shift hours per day, **b** Perceived stress (PSS) by weekly working hours.

A one-way ANOVA showed no significant differences in PSS scores across daily shift durations ($\le 6$, 6–12, $\ge 12$ h; $p = 0.24$), although mean stress levels slightly increased with longer working hours (Fig. 8a). Similarly, another ANOVA assessed weekly working hours (<36, 36, 72, >72 h), with the 72-hour group showing the highest mean stress score ($M = 19.37$; Table 11, Fig. 8b). However, this difference was not statistically significant ($p = 0.619$). Overall, neither daily nor weekly shift duration was significantly associated with perceived stress.

**Table 11:** Mean perceived stress scores by weekly working hours.

Weekly hours	Mean PSS	95% CI
<36 h	17.34	± 1.57
36 h	17.96	± 1.73
72 h	19.37	± 2.38
>72 h	17.53	± 1.94

### Stress predicting negative affect

A linear regression assessed predictors of PANAS Negative Affect scores (Table 12). Perceived stress (PSS) significantly predicted higher negative affect ($\beta$ = 0.325, $p < 0.001$). Mental health history also showed a significant association ($\beta$ = 2.18, $p = 0.036$), while occupational injury was non-significant ($\beta$ = 2.07, $p = 0.129$). The model intercept was 16.14 ($p < 0.001$). These results indicate that perceived stress strongly contributes to negative emotional states, beyond prior mental or injury history.

**Table 12:** Regression analysis predicting negative affect (NA) from perceived stress and health variables.

Predictor	Coefficient ($\beta$)	p-value	Interpretation
Perceived Stress (PSS)	+0.325	<0.001	Higher stress predicts a more negative mood
Mental Health History	+2.18	0.036	Independently associated with higher NA
Injury	+2.07	0.129	Not statistically significant
Intercept	16.14	<0.001	Baseline NA score

### Multivariate predictors of perceived stress

A multiple linear regression was conducted using gender, age, marital status, smoking, drug use, and body weight as predictors of Perceived Stress Scale (PSS) scores (Table 13; n = 200). Age emerged as the strongest predictor: participants aged 26–45 ($\beta = 3.09$, $p = 0.011$) and over 45 ($\beta = 7.95$, $p = 0.041$) reported significantly higher stress than those aged 18–25. Male gender showed a borderline-significant association with lower stress ($\beta = -2.60$, $p = 0.064$). Body weight showed a weak, marginal association ($\beta = 0.065$, $p = 0.085$). Smoking status ($\beta = -4.94$, $p = 0.152$), marital status, and drug use were not statistically significant.Subsections 4.3 to 4.9 visualize the relationships between PSS scores and key demographic and lifestyle variables. These graphical trends support the regression model’s findings, particularly age as the dominant factor in stress variation. The results underscore the importance of age-specific, occupation-sensitive mental health interventions. The standardized beta coefficients indicate small-to-moderate effect sizes, suggesting that while statistically significant, demographic factors such as age exert a measurable but not overwhelming influence on stress levels in practical terms.

**Table 13:** Multivariate regression analysis of predictors of perceived stress.

Predictor	Coefficient ($\beta$)	p-value	Interpretation
Age 26–45 (vs. 18–25)	+3.09	0.011	Significantly higher stress
Age>45 (vs. 18–25)	+7.95	0.041	Highest stress significant
Male (vs. Female)	–2.60	0.064	Lower stress (borderline significant)
Smoker (vs. Ex-smoker)	–4.94	0.152	Lower stress, not significant
Weight (kg)	+0.065	0.085	Weak positive trend (marginal)

## Discussion

This study examined perceived stress, emotional affect, insomnia vulnerability, and shift work-related dysfunction among healthcare professionals in Pakistan. Using validated instruments and multivariate modeling, we identified key demographic and behavioral correlates of stress in a sample of 200 respondents. Results highlighted the influence of age and shift-related variables on psychological well-being in a resource-constrained healthcare system. Moderate stress and insomnia scores on the Perceived Stress Scale (PSS) and Ford Insomnia Response to Stress Test (FIRST) are consistent with prior studies showing elevated distress among healthcare workers in low- and middle-income countries (LMICs), often linked to excessive workload, limited staffing, and inadequate institutional support. Elevated FIRST scores suggest vulnerability to stress-related sleep disturbances, which are known to precede mood instability, cognitive decline, and burnout. A subset of participants also showed symptoms of shift work disorder, as measured by the Shift Work Disorder Index (SWDI), reinforcing evidence that irregular work schedules negatively affect sleep and occupational performance. Because both FIRST and SWDI are self-reported, sleep-related outcomes may reflect subjective perceptions rather than physiological disturbances. Future work should incorporate objective measures such as actigraphy or polysomnography. Despite these burdens, positive affect scores exceeded negative ones on the Positive and Negative Affect Schedule (PANAS), indicating some degree of emotional resilience among healthcare workers. Age was the strongest predictor of stress. Professionals aged 26–45 and those over 45 reported significantly higher stress than younger colleagues. This trend supports earlier findings that mid-career individuals face increasing demands both professionally and personally. The oldest group reported the highest stress overall, possibly due to long-term occupational exposure or reduced coping flexibility. This is especially relevant in LMIC settings where career advancement often brings more responsibility without additional support. These observations support the need for age-focused interventions, particularly for mid- and late-career professionals. These findings should also be interpreted within Pakistan’s collectivist social framework, where interconnected family structures and community obligations play a central role in shaping emotional and occupational experiences. In such contexts, mid- and late-career professionals often bear dual responsibilities, providing leadership and mentorship at work while also fulfilling extensive family duties. These overlapping social roles can intensify the experience of stress, particularly when institutional support and work-life boundaries are weak. Moreover, cultural values that emphasize endurance, modesty, and emotional restraint may discourage open communication about psychological distress, leading many healthcare workers to internalize stress rather than seek professional help. This dynamic partly explains why age remained a stronger predictor of stress than gender or shift type in our analyses, as accumulated social and professional expectations compound over time. Workplace culture further interacts with these cultural norms. In hierarchical healthcare environments, senior staff are often expected to manage administrative loads, mentor junior colleagues, and maintain clinical efficiency despite limited organizational support. These cumulative pressures, combined with family obligations and societal expectations, create a multifaceted stress landscape that extends beyond occupational strain. Understanding stress within this sociocultural framework is therefore essential for designing interventions that resonate with local realities rather than replicating Western-centric approaches. Although female participants reported slightly higher stress levels than males, the difference was not statistically significant. This contrasts with other research suggesting that women in healthcare experience more stress due to dual roles. Contextual differences, such as job roles or cultural expectations, may explain this discrepancy. It is also possible that male participants underreported stress. Future research should examine intersectional factors such as marital strain, childcare responsibilities, and workplace hierarchies that may compound gender-related stress in clinical settings. Married individuals had higher stress than singles, which aligns with prior literature suggesting that family responsibilities add to work-related stress. Widowed participants showed similar stress levels to married individuals, though the small sample warrants cautious interpretation. Passive smokers reported the highest stress, while current smokers had the lowest. Although unexpected, this finding is consistent with literature indicating that some individuals use smoking as a coping mechanism. However, small subgroup sizes and unmeasured factors like social support limit strong conclusions. Drug use, excluding tea, showed no significant association with stress. Higher stress among tea consumers is likely due to widespread tea use in South Asia rather than any direct physiological cause. Shift type and working hours did not significantly predict stress, despite trends suggesting increased stress among night and double-shift workers. Although night and double shifts showed trends toward higher stress, these differences did not reach statistical significance. This may reflect adaptive coping among workers accustomed to irregular schedules, or the buffering effects of institutional support, peer relationships, and self-selection into demanding roles. Alternatively, subgroup sizes may have been too small to detect subtle effects. Future studies with larger samples and detailed workload measures could clarify these patterns and quantify the impact of shift rotation intensity. Participants with 72-hour workweeks reported the highest stress levels. These patterns support existing evidence that long work hours and disrupted sleep cycles contribute to psychological strain. The lack of statistical significance may be due to limited sample size or differences in personal adaptability. Future studies should consider more detailed measures of workload and recovery to better assess these effects. Regression analysis confirmed that stress significantly predicted negative affect, even after accounting for prior mental health history and occupational injury. This affirms the strong connection between chronic stress and emotional difficulties. Mental health history also independently predicted negative affect, underscoring the importance of early psychological assessment and intervention. In multivariate analysis, age remained the most consistent predictor of stress. Gender and body weight showed borderline associations. Other factors, such as smoking status, marital status, and general drug use, were not significant after adjusting for confounders. This suggests that broader structural and occupational influences may have a stronger impact on psychological health than individual lifestyle choices. The study’s strengths include the use of validated psychometric tools and a diverse participant sample, enhancing generalizability to other LMIC healthcare settings. Multivariate analysis enabled adjustment for confounders and clearer identification of independent predictors. The interpretation of these findings should be viewed within the study’s methodological boundaries. However, the study’s limitations include its cross-sectional design, reliance on self-reports, and small subgroups for smoking and drug use. Given the cross-sectional design, causal relationships between shift patterns, stress, and health outcomes cannot be inferred. Longitudinal studies are recommended to examine temporal effects. The absence of physiological or sleep biomarkers also limits the depth of explanation. These findings have practical relevance. Stress reduction programs should focus on mid-career staff and incorporate routine screening and mental health support. Integrating cultural and organizational awareness into occupational stress management is crucial for sustainable well-being among healthcare workers in Pakistan. Interventions should account for collectivist values by fostering peer-support networks and incorporating family-oriented resilience programs that align with local norms of interdependence and shared responsibility. For mid- and late-career professionals, policies promoting flexible scheduling, distributed leadership responsibilities, and institutional recognition of psychosocial well-being can help mitigate cumulative stress. Embedding stress-screening and counseling services within hospital systems, and normalizing their use through leadership endorsement, may further reduce stigma and enhance long-term retention of experienced staff.Adjusting work schedules to reduce night and extended shifts may help reduce long-term stress, even if immediate effects are not apparent. Future research should adopt longitudinal methods and include objective health data to complement self-reports. Additional focus on gender-based coping and the role of organizational culture may also improve understanding of stress in healthcare environments. While these findings contribute to the global understanding of healthcare stress, their applicability remains specific to the Pakistani healthcare context and comparable LMIC environments. Although the conceptual framework included a hypothesis on shift-related cognitive performance, objective data were not collected in this study. This limitation is acknowledged as a direction for future research employing neurocognitive or reaction-time testing.

## Conclusion

This study provides a comprehensive assessment of perceived stress, emotional affect, insomnia vulnerability, and shift work-related dysfunction among healthcare professionals working under shift-based schedules in Pakistan. Using validated psychometric instruments and multivariate statistical modeling, we identified age as the most robust and consistent predictor of stress. Healthcare workers in the 26–45 and>45 age groups reported significantly higher stress than their younger counterparts, underscoring the heightened psychological burden borne by mid- and late-career professionals. Although gender differences in stress were not statistically significant, female participants reported slightly higher levels of stress. Marital status and passive smoking were associated with elevated stress in unadjusted analyses, while body weight exhibited a weak positive association with stress in adjusted models. Despite observable trends, neither shift type nor work hours were statistically significant predictors of stress, suggesting that other contextual and individual factors may moderate the impact of occupational scheduling on psychological health. Perceived stress was a significant predictor of negative affect, independent of mental health history and occupational injury, reinforcing the interconnectedness of stress and emotional functioning in high-pressure clinical environments. Taken together, these findings highlight the need for targeted interventions that address the mental health needs of healthcare professionals, particularly those in mid-career and shift-based roles. Workplace policies that promote stress management and sleep hygiene, alongside accessible mental health support services, are essential for sustaining a resilient and effective healthcare workforce in resource-constrained settings. In practical terms, institutions could implement routine mental-health screening, flexible scheduling to limit consecutive night shifts, and structured resilience or peer-support programs for mid- and late-career professionals. Beyond Pakistan, these results have implications for LMIC healthcare systems where similar resource constraints and workforce dynamics exist, emphasizing the need for national mental health frameworks that integrate occupational stress monitoring into public health policy.

## Data Availability

The datasets generated and analyzed during the current study are available from the corresponding author on reasonable request.
